# *Salmonella enterica* Serovar Napoli Infection in Italy from 2000 to 2013: Spatial and Spatio-Temporal Analysis of Cases Distribution and the Effect of Human and Animal Density on the Risk of Infection

**DOI:** 10.1371/journal.pone.0142419

**Published:** 2015-11-11

**Authors:** Caterina Graziani, Ida Luzzi, Slawomir Owczarek, Anna Maria Dionisi, Luca Busani

**Affiliations:** 1 Department of Veterinary Public Health and Food Safety, Istituto Superiore di Sanità, Rome, Italy; 2 Department of Infectious, Parasitic and Immune-mediated Diseases, Istituto Superiore di Sanità, Rome, Italy; University of Osnabrueck, GERMANY

## Abstract

**Background:**

*Salmonella* Napoli is uncommon in Europe. In Italy however, it has been growing in importance since 2000. To date, no risk factors have been identified to account for its rise. This study aims at describing the epidemiology, spatial and spatio-temporal patterns of *S*. Napoli in Italy from 2000 to 2013, and to explore the role of several environmental correlates, namely urbanization, altitude and number of livestock farms, on the risk of S. Napoli infection among humans.

**Method:**

Data were obtained from Enter-Net Italy, a network of diagnostic laboratories. The data were aggregated at the municipality level. Descriptive epidemiology, multivariate regression models, spatial and spatio-temporal analyses were performed on the number of cases and incidence rates.

**Results:**

*S*. Napoli showed an expanding trend at the national level, and an increasing number of cases. Compared to the other main serovars in Italy, the risk of *S*. Napoli infection was higher in the age group <1 year, and lower in the other age groups. Although urbanization and the number of farms were associated with the risk of *S*. Napoli infection to some extent, their role in the epidemiology of the disease remains inconclusive. *S*. Napoli cases showed a positive global spatial autocorrelation as well as a significant spatio-temporal interaction. Twenty-four spatial and spatio-temporal clusters were identified, seven purely spatial and 17 spatio-temporal, mainly in north-western Italy. Most of the clusters were in areas characterized by urban and industrial settlements surrounded by agricultural land and an abundance of freshwater bodies.

**Conclusions:**

Our results point to the presence, in a number of areas in Italy, of a *Salmonella* of public health concern originating in the environment. This highlights the increasing relevance of environmental, non-food-related sources of human exposure to enteric pathogens.

## Introduction

Nontyphoidal *Salmonella* infections have a significant impact on human health, globally. In Europe, salmonellosis is the second most commonly reported zoonosis [1], [2]. The most frequently reported serovars associated with human illness in the EU are *S*. Enteritidis and *S*. Typhimurium (41.3% and 22.1%, respectively). Recently however, there has been a sharp rise in the number of cases due to *S*. Typhimurium 4,[5],12:i:- (7.2%) [1]. The frequency and relative importance of other serovars vary between EU countries and over time. *Salmonella enterica* serovar Napoli is a case in point. This serovar, relatively uncommon in Europe [3], is among the top serovars causing human infection in Italy [4]. The number of *S*. Napoli cases in Italy has been on the rise since 2000, but no foodborne or environmental factors capable of accounting for this trend have thus far been identified despite efforts to that end [3], [5]. Identification of the sources of *Salmonella* infection is important for the development of effective surveillance and prevention measures. Clinical, epidemiological and microbiological information on human cases, integrated with data from the monitoring of domestic animal reservoirs and foods, have been widely used for source attribution of *Salmonella* cases in Europe. This has been limited to the most common *Salmonella* serovars, however [6], [7]. Moreover, while European legislation requires the monitoring of *Salmonella* in poultry, other domestic and wild reservoir animals are sampled and tested only sporadically.

Spatial and spatio-temporal analyses, including scan statistics, are commonly used to analyse surveillance data, from cancer to infectious diseases such as *Salmonella*, in order to detect outbreaks and to explore spatial, temporal and spatio-temporal patterns and trends [8]. This approach has proven useful in describing the disease, detecting areas at relatively high risk and formulating hypotheses regarding the nature of its spatial distribution [9][10].

Another alternative approach to the analysis of case information is the evaluation of area-level associations. Ecological and individual-level studies have established associations between enteric infections and socio-economic indicators [11][12], but other factors, such as the distribution and density of domestic animal populations are likely to play a role in explaining the spatial pattern of salmonellosis.

The first aim of this study was to describe the epidemiology of *Salmonella enterica* serovar Napoli in Italy from 2000 to 2013, and the main differences between this serovar and the other epidemiologically relevant serovars, namely *S*. Typhiumurim and its monophasic variant, and S. Enteritidis. The second aim was to provide insight into environmental correlates that could be associated with the risk of *S*. Napoli infection in humans.

## Materials and Methods

Data sources: data on cases were obtained from the Enter-Net (IT-ENTER-NET) database (in S2 File), which contains information on *Salmonella* isolates from human cases provided by a network of more than 140 clinical microbiology laboratories covering about 65% of the Italian territory [13], [14]. All 67 021 *Salmonella* strains isolated between 2000 and 2013 were included in the study. The following information was extracted for each isolate: the patient's sex, age group (<1, 1–5, 6–14, 15–64, and ≥65 years), municipality of residence (LAU level 2, formerly NUTS level 5), and date of *Salmonella* isolation. The municipality of residence for each case of *Salmonella* was geocoded to the municipality centroid, identified by latitude and longitude coordinates.

Information about the municipalities' altitude and degree of urbanization were obtained from the Italian National Institute of Statistics (ISTAT) [15]. These two variables were coded following ISTAT's criteria–that is, for altitude: plain (<300 metres above sea level), hill (300–600 metres a.s.l.) and mountain (>600 metres a.s.l.), and for urbanization: “high” for municipalities with over 500 inhabitants/km^2^ and at least 50,000 inhabitants; “medium” for municipalities with 100–500 inhabitants/km^2^ or at least 50,000 inhabitants; “low” for the remaining municipalities.

The reference human population used for the calculation of the average annual incidence was the 2008 Italian population obtained from ISTAT; only municipalities where at least one case of salmonellosis occurred during the study period were included (3 582 municipalities out of 8 047). The number of farms and farmed animals by species and municipality in 2008 was also obtained from ISTAT.

Risk factor analysis: analysis of specific risk factors for *S*. Napoli infection was done considering the cases of salmonellosis due to *S*. Napoli as “cases”, and those attributable to the other serovars as “controls”, following Domingues et al. [9]. Individual (age and gender) and environmental variables (altitude, urbanization, number of farms and farmed animals by species) were analyzed in univariate and multivariate models, and crude (Mantel-Haenszel) and adjusted (logistic regression) ORs were estimated. Statistical significance was set at 0.05. Data analysis was performed using the STATA software, version 12.1 (StataCorp, College Station, USA).

The distribution of cases was mapped using ISTAT's shapefiles of Italy with administrative boundaries (ED-1950-UTM coordinate system, zone 32 N), the CORINE land cover data, made available by the European Environment Agency (http://www.eea.europa.eu/data-and-maps/data/corine-land-cover-2006-raster-2) and the QGIS software version 2.0.1-Dufour (Quantum GIS Development Team (2013). Quantum GIS Geographic Information System. Open Source Geospatial Foundation Project. http://qgis.osgeo.org). To facilitate readability, the Corine map was simplified to include only 7 of the 50 land cover categories identified in Italy. These are: urban and industrial fabric, agricultural land, rice fields, grassland and forests, coniferous forests, glaciers and marshes, and water bodies. The category "rice fields" was maintained because, in northern Italy, the vast areas dedicated to this crop are regularly flooded. The category “coniferous forests” was included as a proxy for altitude, since in Italy, coniferous forests grow between 1000 and 2000 metres a.s.l.

Spatial and spatio-temporal analysis: the spatial distribution of *S*. Napoli cases was explored to identify spatial autocorrelation using the global Moran's I statistics [16]. The crude incidence of *S*. Napoli for the purposes of autocorrelation estimates was calculated at the municipality level. Weighting was based on the inverse distance between centroids. The general spatio-temporal interaction between *S*. Napoli cases was explored using Knox’s statistics [17]. The spatial reference parameter used for the analysis was arbitrarily set to a 25 km radius, so as to take local conditions into account, and reduce environmental fragmentation within clusters. The temporal window was set at 90 days, a period of time sufficient for the infection to be transmitted, the disease to develop and the case to be detected.

The detection of purely spatial and spatio-temporal clusters of *S*. Napoli in Italy in the period 2000–2013 was performed using the SaTScan software, version 9.2 [18][19]. The number of *S*. Napoli isolates was aggregated to the municipality of residence level and assessed for spatio-temporal clustering through retrospective space-time analyses using the discrete Poisson model [18][19].

The Kulldorff scan method was applied to detect spatial aggregates of events (cases) over time, adjusting for the underlying population at risk. Under the null hypothesis, the expected number of cases is proportional to the population size, while the alternative hypothesis is that the number of cases is higher within, than outside the defined spatio-temporal window. For the purpose of the analysis, we used two different populations: the human population of 2008, and the total number of *Salmonella* isolates in the municipality. Here, too, the maximum temporal window was set at 90 days, while the maximum spatial window was set to a 25km radius.

## Results

A total of 1 527 (2.3%) isolates of *Salmonella* serovar Napoli out of 67 021 *Salmonella* isolates were reported from 2000 to 2013.

**Fig 1:**
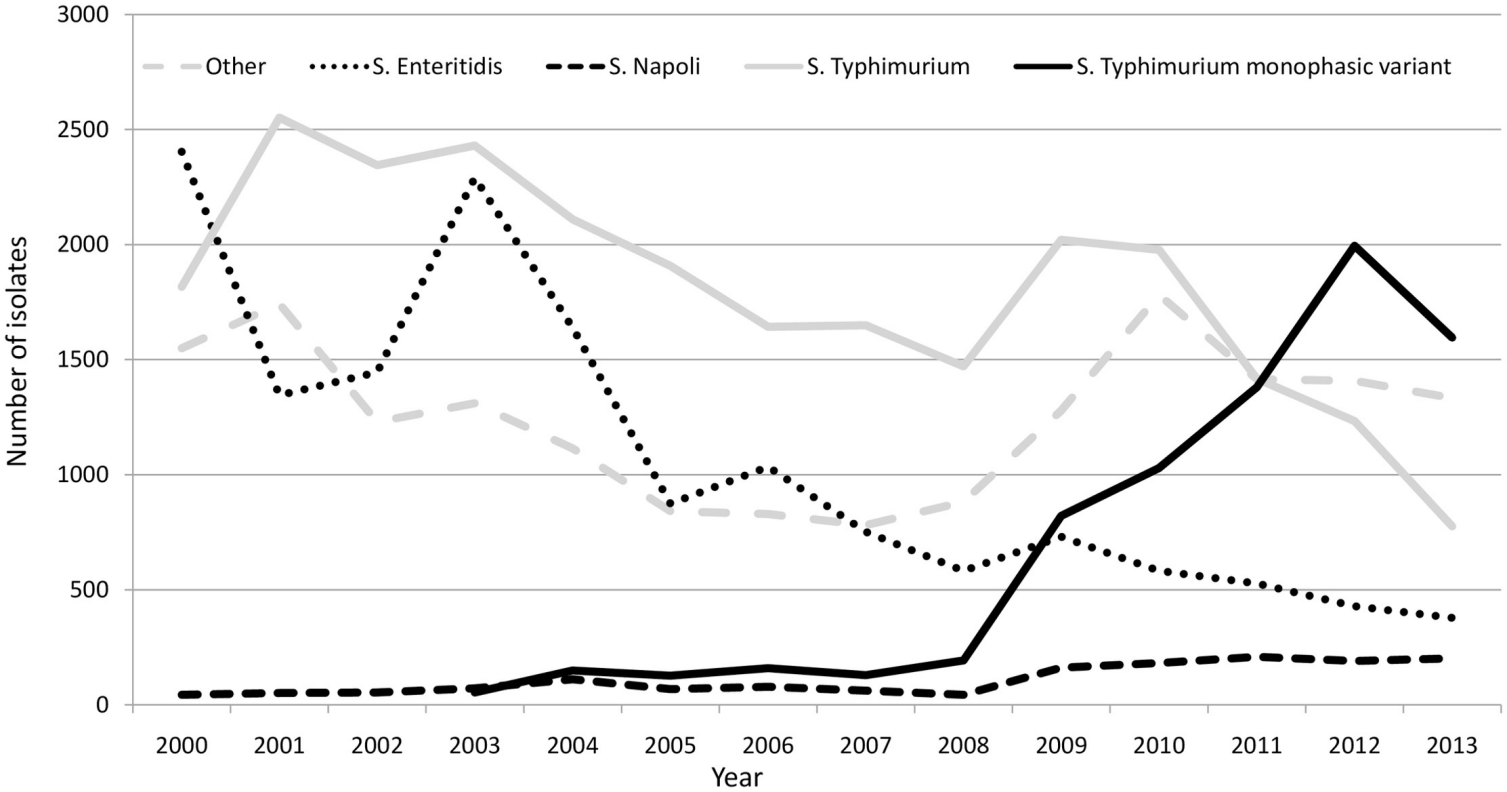
Trends in the number of cases of the main *Salmonella enterica* serovars causing human cases in Italy, 2000–2013

Overall,the annual number of *Salmonella* isolates, which had been constant at an average of 5 500 isolates per year from 2000 to 2004, declined to 4 700 isolates per year between 2005 and 2009, and then rose again from 2010 to 2013 to reach an average of 5 000 isolates per year (Fig 1).

Information on gender was available in 63 403 of the records (94.6%). The proportions of males and females were 51.6% and 48.4%, respectively. The age of the patient was available for 1 387 (90.8%) of the *S*. Napoli cases, and 55 466 (82.8%) of *Salmonella* cases due to other serovars (Table 1).

**Table 1:** Comparison of percentage age distributions across *Salmonella* serovars. Italy, 2000–2013.

Serovar	<1 year	1–5 years	6–14 years*	15–64 years	≥65years	Total (100%)
*S*. Napoli	6.8%	44.7%	16.3%	14.9%	17.3%	1387
*S*. Typhimurium	2.9%	46.1%	19.0%	21.1%	10.9%	21033
*S*. Enteritidis	2.7%	29.0%	20.1%	38.0%	10.2%	12064
*S*. 4,[5],12:i-	3.4%	45.7%	20.2%	16.8%	14.0%	6841
Other serovars	4.4%	28.3%	12.3%	33.2%	21.8%	14141

* not statistically significant (p>0.05)

The municipality of residence was available in 37 058 records (933 *S*. Napoli cases). This was thus the subset of the data used in analyses requiring spatial, population or environmental information.

The average annual incidence of salmonellosis from all serovars was highest among 1–5 year-olds (58.6 cases/100 000), followed by infants under one year of age (26.8/100 000) and children 6–14 years old (15/100 000). In the older age groups: 15–64 and ≥65, the annual average incidence was 3/100 000 and 5/100 000, respectively.

**Fig 2:**
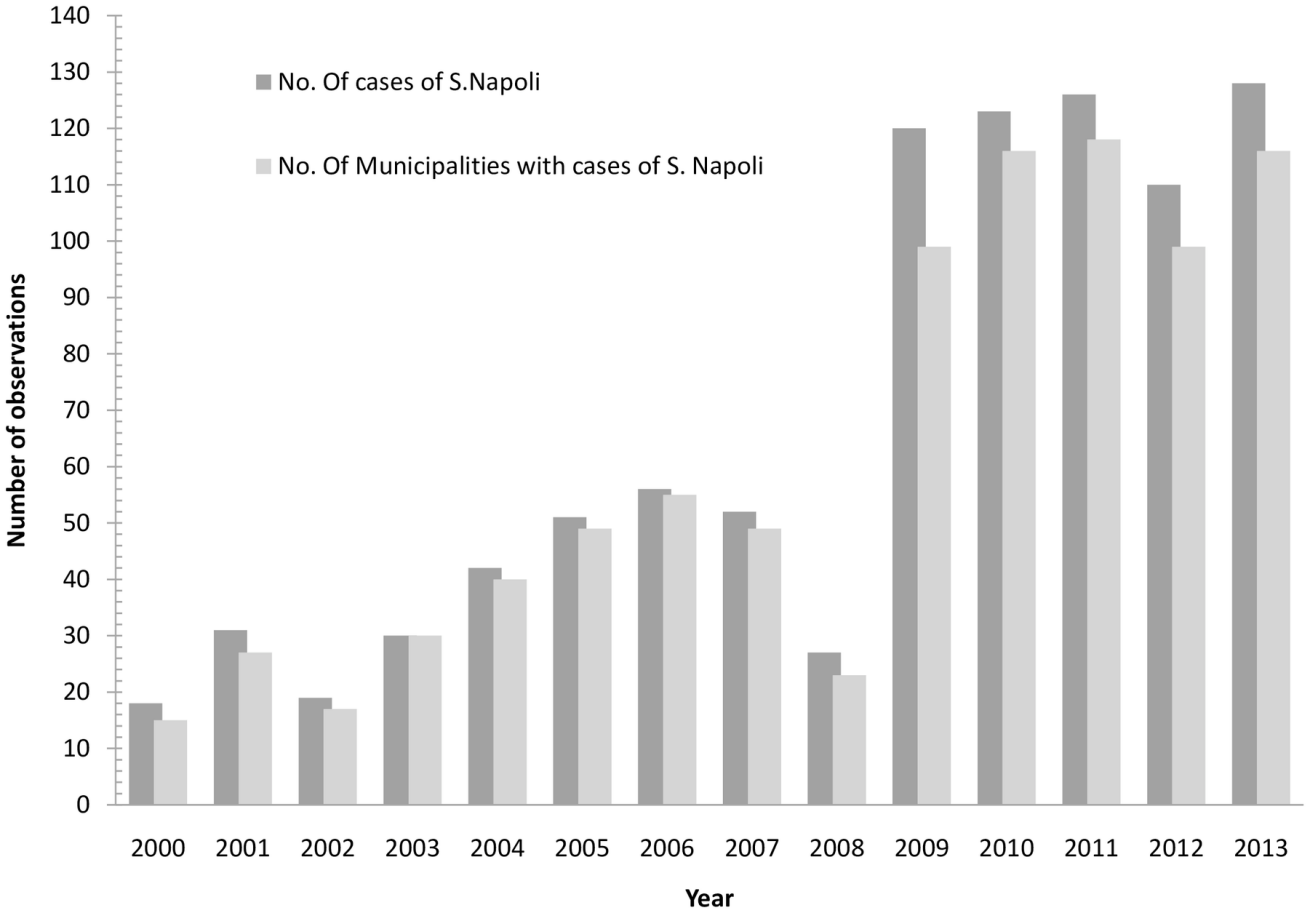
Number of cases of *S*. Napoli and number of municipalities where cases occurred by year. Italy, 2000–2013.

The number of *S*. Napoli cases rose in the period under consideration, from 44 isolates reported in 2000 (0.8% of the total number of Salmonella isolates in that year), to 201 (4.7%) isolates in the last year of the study (Fig 2 and S1 File).

We observed no significant differences between cases due to *S*. Napoli and those due to other serovars (controls) in terms of the proportions of males (53.9% of cases and 51.6% of controls) and females (p = 0.08 by the chi square test).

The age distribution of *S*. Napoli (Table 1) showed 51.5% of cases to have occurred among children up to the age of five, with 6.8% of cases occurring in infants under one year of age. The frequency of cases in the remaining age groups ranged between 14.9% (15–64 year-olds) and 17.3% (≥65 year-olds). Comparing the age distributions across serovars, we found a significantly higher proportion of S. Napoli in infants under one year of age, children 1–5 years old, and elderly individuals ≥65 (p<0.001 by the chi square test). The proportion of 6–14 year-old cases was similar across serovars (p = 0.2 by the chi square test), while the proportion of 15–64 year-olds was significantly lower among *S*. Napoli cases than among cases due to other serovars (p<0.01 by the chi square test) (Table 1).

The proportion of hospitalizations was slightly higher for *S*. Napoli cases than for controls (33.0% vs 29.6%), although information on this variable was unavailable in over 30% of the records for both cases and controls.

The number of municipalities with cases of *S*. Napoli increased dramatically from 2000 to 2013 (Fig 2 and S1 File). In 2000, cases were detected in 18 municipalities, mainly in the north and centre of the country, while in 2013, the number of municipalities in which cases were found was 128, distributed all through the country. Cases of *S*. Napoli were observed throughout the study period (i.e, in more than six out of the 14 years under study), in seven municipalities, mainly in the north-west and centre of the country. In the remaining municipalities, cases occurred sporadically, in one or two years.

The multivariate regression model comparing cases of *S*. Napoli to controls infected with other Salmonella serovars (Table 2) showed no difference in the risk of infection between males and females, but confirmed a higher risk of *S*. Napoli infection than of other *Salmonella* infections for infants, (<1 y/o), as compared to other age groups (OR<1 and p<0.01 for the age groups between 1 year and 64; reference group: ≥65). A rise in the risk of *S*. Napoli, as compared to other serovars, was observed over time: from 2000–2004 (reference category: OR = 1) to 2005–2009 (OR = 2.01, 95%CI = 1.55–2.62), with the highest risk in the final period 2010–2013 (OR = 3.48, 95%CI = 2.69–4.51).

**Table 2:** Multivariate regression model for the association between putative risk factors and the risk of *S*. Napoli, comparing cases of *S*. Napoli to controls infected with other *Salmonella* serovars. Italy, 2000–2013.

Risk factors	Adjusted OR	P value	95%CI
*Gender*			
Male	1.00		
Female	0.89	0.24	0.74–1.08
*Age*			
≥65 years of age	1.00		
< 1 year of age	**1.86**	**0.02**	1.26–2.75
1–5 years of age	0.89	0.35	0.69–1.14
6–14 years of age	**0.63**	**0.04**	0.46–0.86
15–64 years of age	**0.42**	**<0.01**	0.30–0.59
*Period of* Salmonella *isolation*			
2000–2004	1.00		
2005–2009	**2.01**	**<0.01**	1.55–2.62
2010–2013	**3.48**	**<0.01**	2.69–4.51
*Municipality of residence*: *degree of urbanization*			
Low	1.00		
Medium	**1.64**	**<0.01**	1.22–2.21
High	1.00	0.99	0.68–1.49
*Municipality of residence*: *altitude*			
>600 metres a.s.l (mountains)	1.00		
300–600 metres a.s.l (hills)	1.13	0.37	0.86–1.49
<300 metres a.s.l (plain)	1.15	0.34	0.86–1.54
*Municipality of residence*: *number of livestock farms*			
Cattle	**0.99**	**<0.01**	0.99–1.00
Pig	0.99	0.45	0.98–1.01
Goat and sheep	1.00	0.75	0.99–1.00
Poultry	1.00	0.99	0.98–1.02
Other species	1.00	0.50	0.99–1.01

In bold: statistically significant OR (p<0.05)

Altitude was not associated with the risk of *S*. Napoli infection. A medium degree of urbanization, on the other hand, was found to be associated with a higher risk of *S*. Napoli than of other *Salmonella* serovars (OR = 1.64, 95%CI = 1.22–2.21).

The risk of *S*. Napoli infection was not associated with the number of livestock farms in the municipality of residence. This held true regardless of the species reared. The only exception was a weak inverse association between the risk of infection and the number of dairy-cattle farms (OR = 0.99, 95%CI = 0.99–1.00).

*S*. Napoli showed a global positive spatial autocorrelation (Moran’s I index = 0.02, p<0.01), namely that municipalities that were geographically close together exhibited incidence rates that were more similar than municipalities that were further apart. Considering the entire study period, we found a significant spatio-temporal interaction among *S*. Napoli cases (p<0.01 by Knox’s statistic); in other words, cases that clustered together in space tended to cluster in time as well.

**Fig 3:**
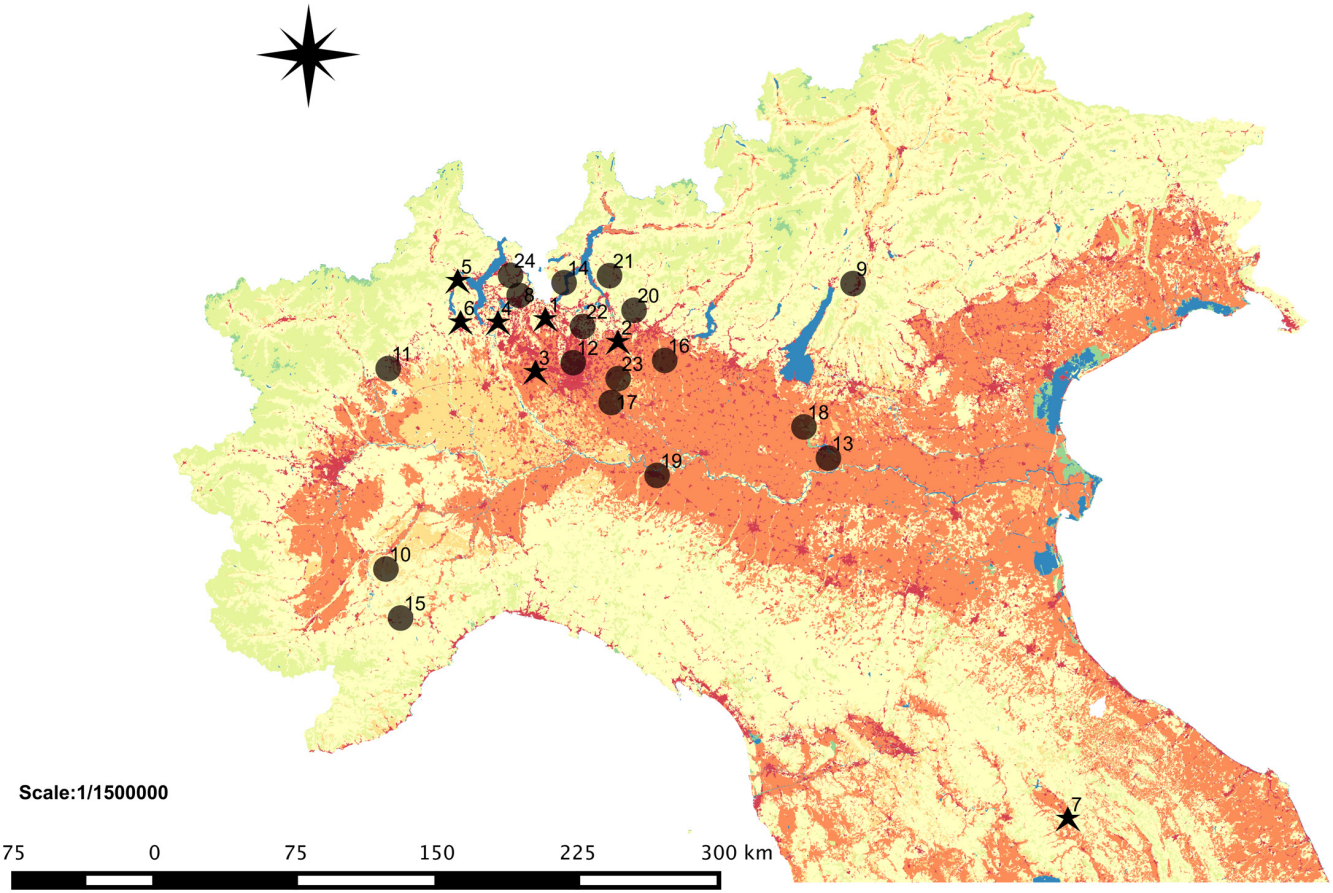
Location of spatial and spatio-temporal clusters of *Salmonella enterica* serovar Napoli human cases in Italy, 2000–2013 (Black stars numbered from 1 to 7 are the centroids of purely spatial clusters; black dots numbered from 8 to 24 are the centroids of spatio-temporal clusters).

Spatial and spatio-temporal cluster analysis detected 24 clusters, seven purely spatial and 17 spatio-temporal (Fig 3, S3 File and Table 3), with 352 cases involved, 74 of them in spatio-temporal clusters. All clusters had radii of <10km, ranging between 3.41 and 9.84km.

**Table 3:** Description of the purely spatial and spatio-temporal clusters in Fig 3.

Cluster ID	Province	Radius (Km)	Start date (dd/mm/yy)	End date (dd/mm/yy)	N. of cases
*Purely spatial clusters*					
1	Como	9.82	-	-	65
2	Monza	9.27	-	-	54
3	Milan	9.77	-	-	52
4	Varese	9.59	-	-	45
5	Verbano	9.84	-	-	27
6	Novara	9.80	-	-	21
7	Perugia	5.00	-	-	14
			-	-	65
*Spatio-temporal clusters*					
8	Varese	5.48	07/07/04	10/08/04	4
9	Trento	5.50	22/09/04	28/09/04	2
10	Cuneo	3.90	06/07/05	27/09/05	3
11	Biella	6.00	04/06/08	10/06/08	2
12	Milan	9.08	17/06/09	18/08/09	11
13	Mantova	9.14	02/09/09	13/10/09	4
14	Como	3.41	21/07/10	05/10/10	3
15	Cuneo	6.50	18/08/10	31/08/10	2
16	Bergamo	8.98	13/07/11	20/09/11	5
17	Lodi	7.87	06/07/11	20/09/11	5
18	Mantova	9.58	24/08/11	18/10/11	4
19	Piacenza	5.50	10/10/12	30/10/12	4
20	Bergamo	7.03	11/07/12	04/09/12	5
21	Lecco	8.21	26/06/13	20/08/13	4
22	Monza	8.06	03/07/13	03/09/13	8
23	Milan	7.40	14/08/13	10/09/13	5
24	Varese	4.62	07/08/13	20/08/13	3

The seven spatial clusters were in Lombardy (four clusters, numbered 1–4, involving a total of 216 cases), in Piedmont (two clusters, numbers 5 and 6, with 27 and 21 cases, respectively) and in Umbria (cluster 7, with 14 cases). As for the 17 spatio-temporal clusters, all were in northern Italy. The highest number of spatio-temporal clusters was observed in Lombardy (12 out of 17), and the remaining five occurred in Piedmont (3), Emilia-Romagna and Trentino Alto Adige. The first spatio-temporal cluster detected occurred in the province of Varese from July to August 2004; the last occurred in the same province in August of 2013. No spatio-temporal clusters were detected from 2000 to 2003. The total number of cases involved in spatio-temporal clusters was 74. The number of cases per cluster ranged from 2 to 11 (median 4). These cases did not differ from unclustered cases with respect to age and sex distributions.

As shown in Fig 3, the clusters, both purely spatial and spatio-temporal, were located in highly fragmented areas, where human settlements (urban and industrial) are surrounded by different types of agricultural lands, woods, semi-natural areas and wetlands (lakes, ponds, rivers, streams).

## Discussion

We investigated the epidemiology of *S*. Napoli in Italy, taking into account environmental features, urbanization settings and livestock density.

*Salmonella* serovar Napoli in Italy represents an enigma. Although a number of epidemiological studies and microbiological analyses have been carried out on human cases and isolates, no single factor has been recognized as a possible explanation for the observed rise.

Indeed, from 2000 onward, while the main serovars *S*. Enteritidis and *S*. Typhimurium decreased, a slight but constant increase in the number of cases of *S*. Napoli was observed in Italy. Compared to the trend of the other emerging serovar, the *S*. Typhimurium monophasic variant 4,[5],12:i-, *S*. Napoli's ascent is moderate, but the monophasic variant has well established reservoirs in livestock that are likely to have favoured its entrance into the food chain, and consequent rapid emergence and dissemination [20]. Foodborne exposure to *S*. Napoli, on the other hand, appears to be negligible [3][5].

Another difference between *S*. Napoli and the other *Salmonella* serovars concerns the proportion of cases in the different age groups, with a significantly greater involvement of young children, possibly due to different exposure patterns. For young children/toddlers, foodborne exposure may be less important than other routes [21], or their different eating habits may have played a role in increasing the risk of *S*. Napoli infection.

Our analysis of the possible role of three area-level risk factors for *S*. Napoli infection–urbanization, altitude and livestock density–yielded inconclusive results. The lack of association with the presence of livestock is consistent with earlier findings suggesting that domestic animals do not serve as reservoir hosts for this serovar.

Wildlife species, on the other hand, have been found to carry *Salmonella*. Indeed, different *Salmonella* serovars, including *S*. Napoli, were isolated from both foxes and wild boar in Lombardy between 2008 and 2010 [22, 23]. Conceivably, the higher risk associated with a medium degree of urbanization in the present study, may be related to a higher degree of human-wildlife (rodents, reptiles, birds, medium-sized and larger carnivores and ruminants) habitat permeability in such areas. The urbanization of wildlife has previously been described as a risk factor for human infection, playing a significant role in the transmission of several zoonotic pathogens (e.g., *Echinococcus multilocularis* and tick-borne infections), but has never been associated with an increased risk of enteric infections [24, 25].

The clusters that we observed were mainly located in areas characterized by fragmented landscapes, with human settlements, urban and industrial, surrounded by agricultural land and an abundance of freshwater bodies (ponds, lakes, rivers and streams). Contact with freshwater bodies (bathing and/or playing in or in the proximity of lakes, ponds and rivers) from previous epidemiological studies carried out in the region of Lombardy was identified as a specific risk factor for *S*. Napoli infection suggesting the existence of environmental *S*. Napoli reservoir [5]. The possible presence of *S*. Napoli reservoirs in the environment is further supported by the results obtained applying different molecular typing techniques to *S*. Napoli isolates collected in Italy between 2000 and 2009 from different sources: human cases, wildlife and surface waters. We observed that isolates originating in the same areas were genetically similar (clones) even if they were isolated from different sources, and/or in different years [26].

With regard to the observed spatio-temporal clusters, we speculate that a number of persistent point-sources of exposure capable of causing small outbreaks and probably related to freshwater and its use for recreational purposes, are present in the area. Drinking water does not seem to be implicated as a source of exposure, since an involvement of water supply systems would likely have resulted in larger outbreaks.

*S*. Napoli has also been isolated from vegetables, and the consumption of fresh leafy vegetables (e.g. rocket salad) linked to outbreaks of this serovar [26]. The relative importance of such exposure was unclear, however, since neither clustering around the production areas (mainly located in southern Italy), nor poor spatial autocorrelation of cases were observed, the latter a characteristic feature of outbreaks related to large scale distribution of food items.

We used laboratory surveillance data on salmonellosis, a valid source of data which, however, usually captures only a small proportion of the real number of cases. Another limitation was the lack of information on the presence of *Salmonella* in potential sources of infection, due to the absence of nationwide monitoring of enteric pathogens in surface water and wild animals. The limited data available on *S*. Napoli isolated from water and wildlife species did not allow us to interpret the role of these factors in the epidemiology of the disease. Furthermore, the range of available area-level information was relatively limited. Harmonized information at the municipality level included only altitude, urbanization and animal farming density. Additional data, such as wildlife composition and density, human populations' behaviour in terms of recreational water use and other relevant practices, environmental and meteorological data, could have provided valuable insights into potential transmission mechanisms and risk factors.

With regard to the observed clustering in north-western Italy this result could be biased by the fact that municipalities tend to be smaller in that area, as compared to central and southern Italy due to the higher population density. Thus, centroids are closer to each other than in the centre or in the south of the country. Still, the finding that *S*. Napoli occurs mostly in northern Italyis supported by the fact that most of the isolates were reported by laboratories from that area even though all of the network's laboratories, nationwide, were repeatedly alerted to pay attention to and notify any cases of *S*. Napoli.

*S*. Napoli is an emerging public health concern in Italy, but key information, essential for the implementation of targeted prevention and control measures, is still lacking. The unique situation in Italy, of a *Salmonella* serovar of environmental origin that ranks among the five most important serovars in the country, deserves attention, especially in the context of the increasing importance of environmental and non-food related sources of human exposure to enteric pathogens [21]. Moreover, the expected changes in climate and their impact on the environment, risk expanding the role of environmental sources of infection, facilitating the spread of enteric pathogens such *S*. Napoli into human populations [27]. We have identified a number of geographical areas where continuous infection of humans occurred. More information is needed however,–specifically regarding the role of the various animal species as reservoir hosts, and potential pathways of human exposure–to gain a clearer picture of the ecology and epidemiology of *S*. Napoli. This would allow us to design targeted control and prevention measures, such as the monitoring of surface waters for recreational use, or information and education campaigns during the summer, which can contribute to reduce the population's risk of exposure.

## Supporting Information

S1 File Maps of the distribution of the *S*. Napoli cases in Italy. (PPTX)

S2 File Complete, anonymized dataset. (ZIP)

S3 File Map of the first and second order of nearest neighbors clustering (big dots and stars are the spatial and spatio-temporal clusters as reported in Fig 3; small dots without numbers are the cluster detected by the first order nearest neighbors analysis; dotted ellipsis is the area identified by the second order nearest neighbors analysis). (PDF)
