# Supplementary material for: Salmonella enterica Serovar Napoli Infection in Italy from 2000 to 2013: Spatial and Spatio-Temporal Analysis of Cases Distribution and the Effect of Human and Animal Density on the Risk of Infection
Source: PLoS One. 2015 Nov 11;10(11):e0142419. doi: 10.1371/journal.pone.0142419 (PMC4641638; doi:10.1371/journal.pone.0142419)
Supplement: S1 File — (PPTX) [file pone.0142419.s001.pptx]

## Slide 1
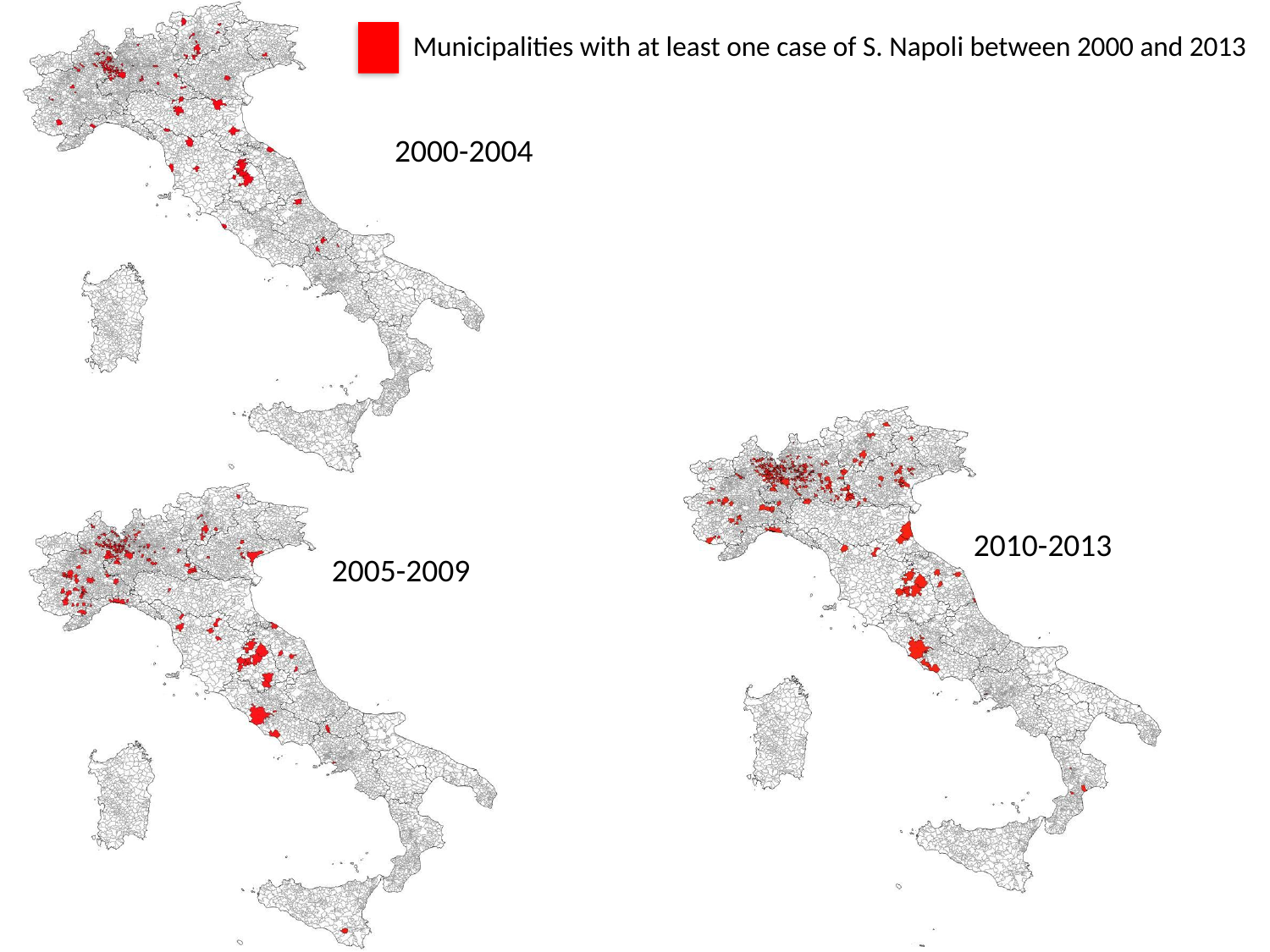

Municipalities with at least one case of S. Napoli between 2000 and 2013
2000-2004
2010-2013
2005-2009

## Slide 2
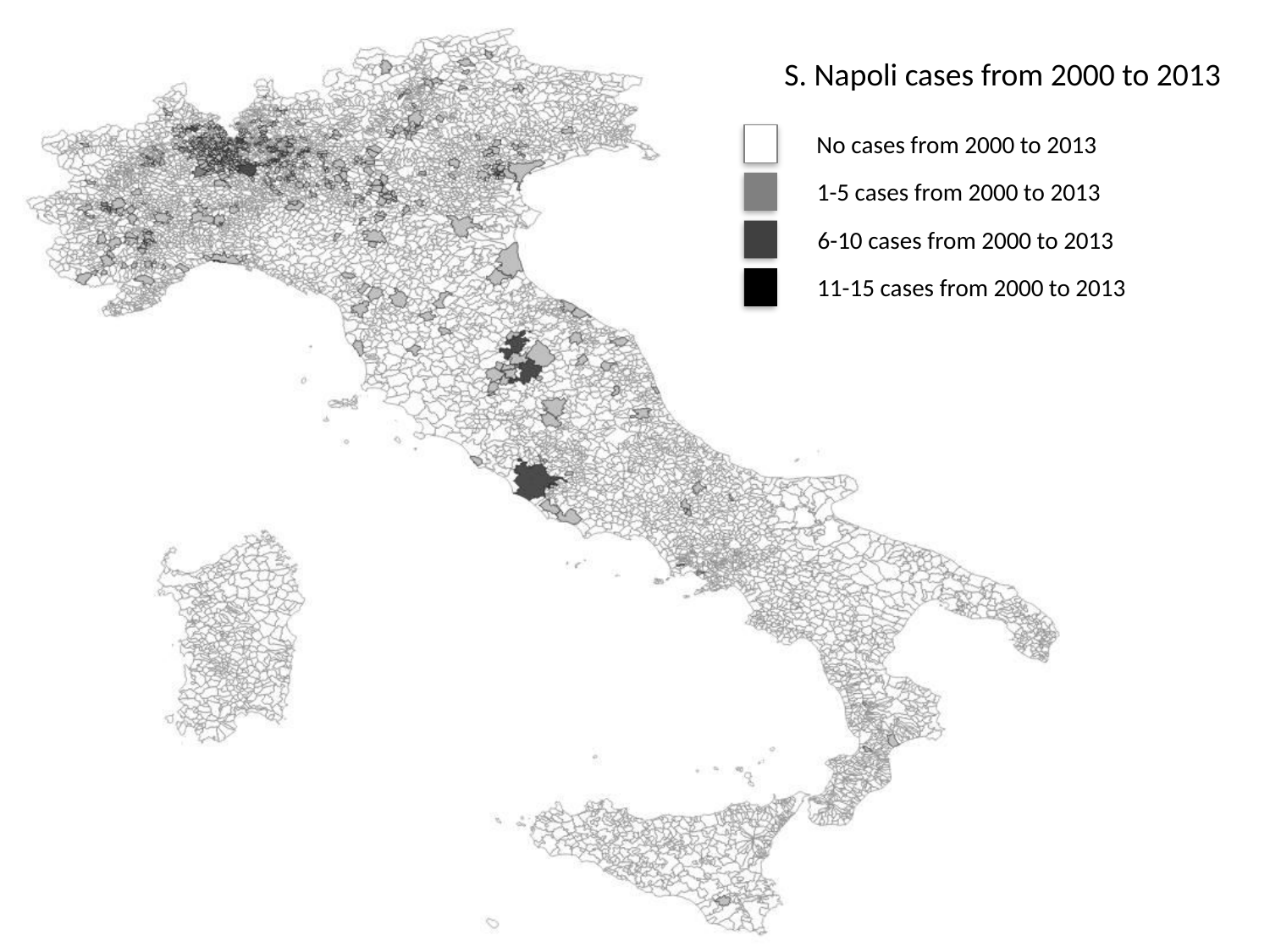

S. Napoli cases from 2000 to 2013
No cases from 2000 to 2013
1-5 cases from 2000 to 2013
6-10 cases from 2000 to 2013
11-15 cases from 2000 to 2013
