# Supplementary figures and images for: Salmonella enterica Serovar Napoli Infection in Italy from 2000 to 2013: Spatial and Spatio-Temporal Analysis of Cases Distribution and the Effect of Human and Animal Density on the Risk of Infection
Source: PLoS One. 2015 Nov 11;10(11):e0142419. doi: 10.1371/journal.pone.0142419 (PMC4641638; doi:10.1371/journal.pone.0142419)

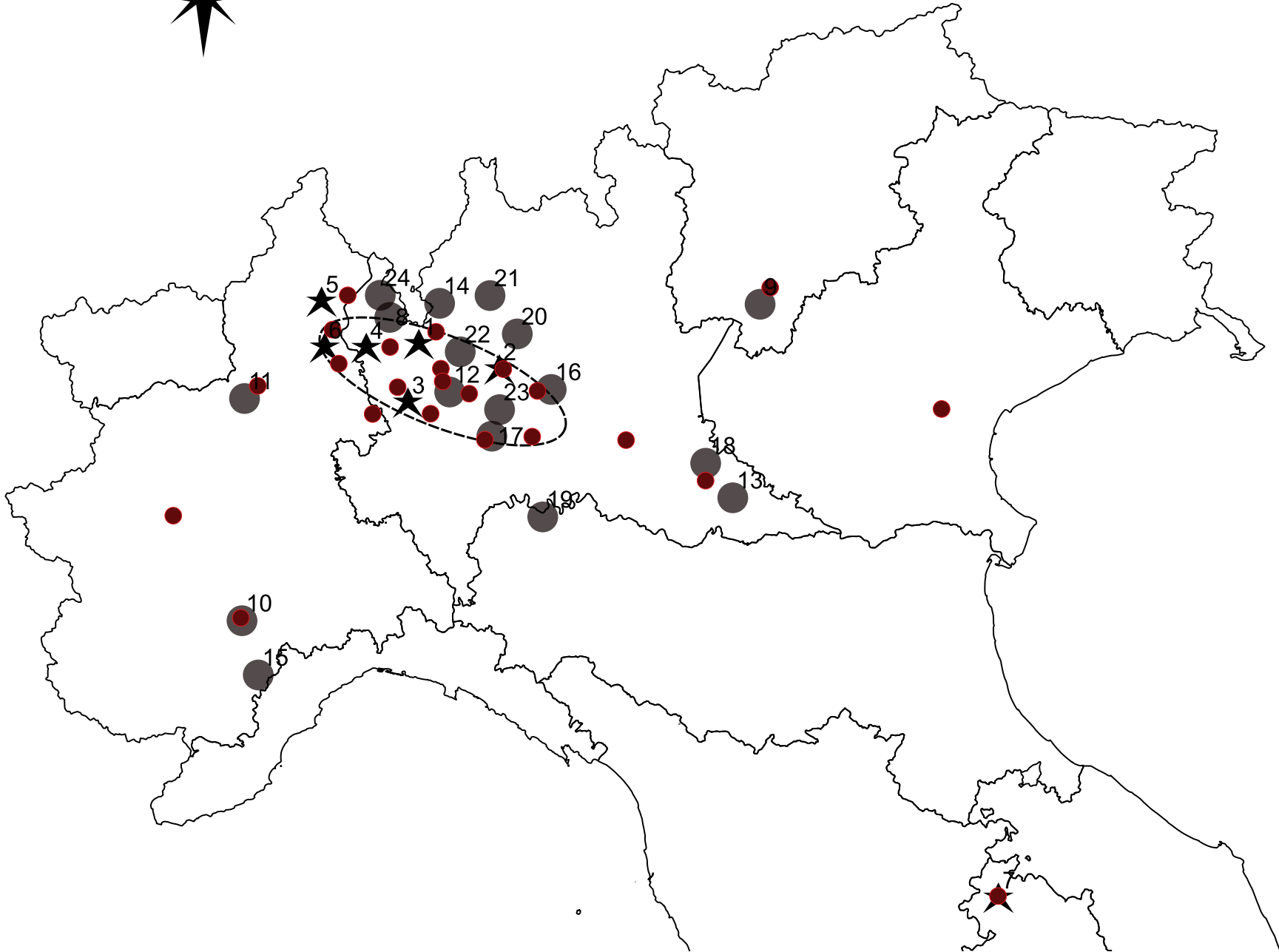

**Scale:1/1500000**

Supplement: S3 File — (PDF) [file pone.0142419.s003.pdf]
